# Bipolar spectrum disorders are associated with increased gray matter volume in the medial orbitofrontal cortex and nucleus accumbens

**DOI:** 10.1002/jcv2.12068

**Published:** 2022-03-08

**Authors:** Katherine S. F. Damme, Lauren B. Alloy, Nicholas J. Kelley, Ann Carroll, Christina B. Young, Jason Chein, Tommy H. Ng, Madison K. Titone, Corinne P. Bart, Robin Nusslock

**Affiliations:** ^1^ Department of Psychology Northwestern University Evanston Illinois USA; ^2^ Department of Psychology Temple University Philadelphia Pennsylvania USA

**Keywords:** bipolar spectrum disorder, frontostriatal, gray matter volume, male, reward, risk

## Abstract

**Objective:**

Elevated sensitivity to rewards prospectively predicts Bipolar Spectrum Disorder (BSD) onset; however, it is unclear whether volumetric abnormalities also reflect BSD risk. BSDs emerge when critical neurodevelopment in frontal and striatal regions occurs in sex‐specific ways. The current paper examined the volume of frontal and striatal brain regions in both individuals with and at risk for a BSD with exploratory analyses examining sex‐specificity.

**Methods:**

One hundred fourteen medication‐free individuals ages 18–27 at low‐risk for BSD (moderate‐reward sensitivity; *N* = 37), at high‐risk without a BSD (high‐reward sensitivity; *N* = 47), or with a BSD (*N* = 30) completed a structural MRI scan of the brain. We examined group differences in gray matter volume in a priori medial orbitofrontal cortex (mOFC) and nucleus accumbens (NAcc) regions‐of‐interest.

**Results:**

The BSD group had enlarged frontostriatal volumes (mOFC, NAcc) compared to low individuals (*d* = 1.01). The mOFC volume in BSD was larger than low‐risk (*d* = 1.01) and the high‐risk groups (*d* = 0.74). This effect was driven by males with a BSD, who showed an enlarged mOFC compared to low (*d* = 1.01) and high‐risk males (*d* = 0.74). Males with a BSD also showed a greater NAcc volume compared to males at low‐risk (*d* = 0.49), but not high‐risk males.

**Conclusions:**

An enlarged frontostriatal volume (averaged mOFC, NAcc) is associated with the presence of a BSD, while subvolumes (mOFC vs. NAcc) showed unique patterning in relation to risk. We report preliminary evidence that sex moderates frontostriatal volume in BSD, highlighting the need for larger longitudinal risk studies examining the role of sex‐specific neurodevelopmental trajectories in emerging BSDs.

## Graphical abstract

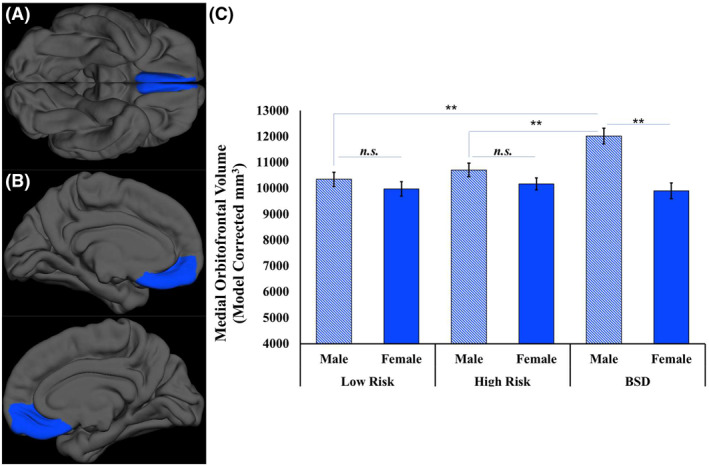

Enlarged volume in the mOFC may be associated with BSD disease processes in males, whereas enlarged NAcc volume may reflect a marker of risk for BSD in males. These findings emphasize the potential role of sex and neurodevelopment in the risk for a BSD. Additionally, these findings highlight the need for larger longitudinal risk studies examining the role of sex‐specific neurodevelopmental trajectories in emerging BSDs.

**Key points**

- Extant research describes bipolar spectrum disorder (BSD) as relating to enlarged frontostriatal volumes; the present study separates risk markers from disease sequalae in a clinical high‐risk model
- Enlarged frontal (mOFC) volumes were associated with disease processes, progression, or onset.
- Volume differences were driven by male participants, and may reflect a sex‐specific pathophysiology that may reflect clinical observations of increased mania in men
- Sex and neuromaturational processes near time of onset may be critical considerations in future risk models of BSDs.

## INTRODUCTION

Bipolar Spectrum Disorders (BSDs) include several disorders (i.e., cyclothymia, bipolar II, and bipolar I disorder) that tend to progress in severity over time (Alloy, Urošević, et al., 2012; Birmaher et al., 2009; Kochman et al., 2005). This tendency to progress in severity highlights the importance of identifying corollaries of risk that can facilitate early detection, clarify pathophysiology, and generate targets for early intervention (Angst et al., 2002; Cassano et al., 1999). Clinical risk models of BSD indicate that elevated self‐reported reward sensitivity prospectively predicts BSD onset (Alloy, Bender, et al., 2012). Additionally, individuals with a BSD tend to show volumetric abnormalities in brain regions that process rewards (Nusslock & Alloy, 2017). However, it is unclear whether such volumetric abnormalities predate the onset of BSD, like self‐reported reward sensitivity (Wise et al., 2017). Understanding neural profiles of risk can provide insights into the pathophysiology of BSD symptoms. Furthermore, BSD symptoms typically emerge in adolescence and early adulthood when sex‐specific trajectories of neurodevelopment in reward‐related regions occur (Bellivier et al., 2003; Giedd et al., 2012; Mills et al., 2016; Paus et al., 2008). Yet, sex rarely is examined in research on BSD risk. The current study used a behavioral high‐risk design to examine volumetric differences in reward‐related neural regions between individuals at low‐risk for BSDs, high‐risk for BSDs but who have not yet developed the illness, and individuals with BSDs. Finally, this paper explores the potential role of sex in these volumetric differences.

The Reward Hypersensitivity Model proposes that risk for a BSD is characterized by a hypersensitivity to rewarding stimuli in the environment (Alloy et al., 2016; Gruber & Johnson, 2009; Johnson, 2005; Johnson et al., 2012; Nusslock & Alloy, 2017; Nusslock et al., 2014). In line with this view, self‐report, behavioral, and neurophysiological data suggest that abnormally elevated reward sensitivity is a risk factor for both the development and progression of BSDs (Alloy & Abramson, 2010; Alloy, Bender, et al., 2012; Alloy et al., 2015; Alloy, Urošević, et al., 2012; Urošević et al., 2008). In fact, Alloy, Urošević, et al. (2012) demonstrated that high reward sensitivity participants had a significantly greater likelihood of developing a first onset of BSD (12.3% absolute risk) over a prospective 1‐year follow‐up. Notably, these conversion rates are comparable to the lifetime conversion rates associated with being a first‐degree relative of a BSD individual (9%; for a review, see Barnett & Smoller, 2009). Among individuals diagnosed with a BSD, heightened reward sensitivity also predicts progression of the illness, including conversion to a more severe form of the disorder (Alloy, Bender, et al., 2012; Nusslock, Harmone‐Jones, et al., 2012), increased manic symptom severity (Meyer et al., 2001), and shortened time to recurrence of a hypo/manic episode (Alloy et al., 2008). Taken together, these studies suggest that elevated reward sensitivity is a marker of risk for both BSDs onset and course.

Extant research also has documented that BSDs are associated with abnormal gray matter volume in reward‐related brain regions, including in both the frontal cortex and the ventral striatum (Abe et al., 2016; DeBello et al., 2004; Ha et al., 2009; Haznedar et al., 2005; Ivleva et al., 2013; Lyoo et al., 2004; McDonald et al., 2004; Rimol et al., 2010, 2012; Wise et al., 2017). These frontal and striatal volume differences have been reported across all BSDs (Abe et al., 2016; DeBello et al., 2004; Haznedar et al., 2005; Rimol et al., 2010, 2012; Strakowski et al., 2002). Among frontal regions, the medial orbitofrontal cortex (mOFC) is associated with BSD risk (Stanfield et al., 2009) and is involved in maintaining current reward value and approach behavior (Kringelbach, 2005). Indeed, bipolar I and II disorder have been associated with an enlarged mOFC volume (Abe et al., 2016; Rimol et al., 2012). Within the striatum, studies report that BSDs are specifically associated with an enlarged nucleus accumbens (NAcc; Rimol et al., 2010), an area involved in reward anticipation and predicting the rewarding value of a stimulus (see Bailiki et al., 2013; Haber & Knutson, 2010). Together, these studies suggest that volumetric differences in reward‐related brain regions are relevant to the pathophysiology of BSDs.

These volumetric differences likely reflect the confluence of many neurodevelopmental processes (e.g., synaptogenesis, synaptic pruning, apoptosis, and myelination; Natu et al., 2019), and thus represent a general marker of BSD, as opposed to any specific cellular processes. Additionally, symptoms of BSDs emerge in a developmental context, late adolescence and early adulthood (Bellivier et al., 2003; Paus et al., 2008). Early adulthood is a critical period for neurodevelopment (Giedd et al., 2012), sex steroids (Shirtcliff et al., 2009), and BSD onset (Carter et al., 2003). This neurodevelopment, in part, reflects sex‐specific endocrine changes (Gennatas et al., 2017; Goddings et al., 2014). These endocrine changes have been linked to depression and mania (Cookson, 1985; Galvan, 2013), with androgens such as testosterone predicting an increased number of manic episodes (Sher et al., 2012). In studies of postpartum mania, high levels of estrogens were related to increased mania and striatal sensitivity to dopamine (Cookson, 1985). The effects of these hormones also may partially account for reported differences in the initial presentation of illness, as men with a BSD tend to present with more mania, and women with high levels of depression (Goodwin & Jamison, 2007; Saunders et al., 2015). Pubertal development also impacts the development of reward‐related brain areas independent of age (Urošević, Collins, Muetzel, Schissel, et al., 2014). Specific hormones also show specific effects, with testosterone being related to higher striatal responsivity in pubertal boys (Forbes et al., 2010). Yet, the potential role of sex and neurodevelopment in the risk for bipolar spectrum disorders remains unclear but should be considered or accounted for in biomarkers of risk for BSDs.

The current study examined gray matter volume in the mOFC and NAcc, a subnucleus of the ventral striatum, using a behavioral high‐risk design that included individuals at low‐risk for BSDs (moderate‐reward sensitivity and no BSD diagnosis), high‐risk for BSDs (high reward sensitivity and no BSD diagnosis), and individuals with a BSD (high reward sensitivity and a BSD diagnosis; see Nusslock & Alloy, 2017 for review). In line with previous research, we hypothesized that BSD participants would have enlarged mOFC and NAcc volumes. Extending previous work, our high‐risk design allowed us to distinguish whether these expected volume differences in BSD are a marker of risk for BSD or uniquely associated with the onset of BSDs, using the risk logic described by Wiggins et al. (2017). If only BSD participants display frontal and/or NAcc enlargement, then such volumetric differences may reflect disease processes rather than a pre‐existent marker of risk. Alternatively, if the high‐risk group also displays volume enlargement, this may reflect a pre‐existent risk for the illness. Finally, we conducted exploratory analyses examining both sex and age as moderators of the relationship between frontostriatal volume and BSD group status.

## MATERIALS AND METHODS

### High‐risk participant recruitment

Participants were recruited from a large longitudinal investigation of BSDs. Participants were recruited into this longitudinal study via a two‐stage selection procedure (Alloy, Urošević, et al., 2012). In the first stage, 9991 students (ages 14–19) from the Philadelphia area completed two measures: the Behavioral Inhibition System/Behavioral Activation System scales (BIS/BAS; Carver & White, 1994) and the Sensitivity to Punishment/Sensitivity to Reward Questionnaire (SPSRQ; Torrubia et al., 2001). Participants scoring in the 40–60th percentile on both the BAS‐Total subscale of the BIS/BAS scales and the reward subscale of the SPSRQ were classified as having moderate (i.e., normative levels) reward sensitivity and considered at low‐risk for BSDs (*n* = 750). Participants scoring in the 85–100th percentile on both of these measures were classified as having high reward sensitivity and considered at high‐risk for BSDs (*n* = 1200). These percentiles were validated in prior studies (Alloy, Urošević, et al., 2012). From this initial screening, 539 individuals (334 high reward/high‐risk and 205 moderate reward/low‐risk) returned for diagnostic screening. In this second stage of screening, participants completed a semi‐structured diagnostic interview using the expanded Schedule for Affective Disorders and Schizophrenia‐ Lifetime interview (exp‐SADS‐L; Alloy, Urošević, et al., 2012; Endicott & Spitzer, 1978). Based on the exp‐SADS‐L, we further classified participants according to whether they had a lifetime BSD. All BSD individuals were high reward sensitivity individuals, and no individuals in the low or moderate reward sensitivity groups were diagnosed with a BSD based on the exp‐SADS‐L. We also used the exp‐SADS‐L to exclude individuals with a primary psychotic disorder. The imaging session was offered to individuals as an optional additional research session, and diagnostic/risk group was confirmed on the day of the scan.

### Interpreting risk group differences

Group differences were interpreted using the nosology described by Wiggins et al. (2017). Effects that appeared in the high‐risk and BSD groups but not in the low‐risk group were considered markers of risk that predated the onset of the illness. Effects that were specific to the BSD group (i.e., not observed in either the low‐ or high‐risk groups) were considered a corollary of the disease, meaning they are associated with some aspect of the disease process, onset, or progression, rather than reflecting a preexisting risk factor. In the case of an inconclusive finding where the BSD group significantly differs from low‐risk individuals, but the high‐risk group differs from neither and displays intermediary effects, the heterogeneity of the intermediate group will be discussed.

### Participants

One hundred and thirty right‐handed young adults from the larger project completed a single MRI session (Table 1). After excluding individuals who were taking psychiatric medications at the time of the MRI scan, our final analytic sample for the present paper was 114 participants (52% female; mean age = 20.71; age range 18%–27%; 42% non‐Caucasian, see Table S1). No individuals were excluded due to the quality of the MRI data following the quality control procedures laid out in Beckenhausen et al. (2016). This included 37 low‐risk individuals (moderate reward sensitivity), 47 high‐risk individuals (high reward sensitivity), and 30 individuals with a BSD (high reward sensitivity plus a BSD; 8 bipolar not otherwise specified, 3 cyclothymia, 15 bipolar II, 4 bipolar I), Table 1. See supporting information including Table S3 for analyses adjusting for comorbidity. All participants were free of any current psychiatric medication use (Lyoo et al., 2010; Moore et al., 2009; Sassi et al., 2002). Participants provided informed written consent and were compensated. The IRB at Temple University approved all study protocols.

**TABLE 1:** Demographics by group

Demographics	Low‐risk	High‐risk	BSD	Total	Group comparison	*p*‐value
	*n* = 37	*n* = 47	*n* = 30	*n* = 114		
	*M* (SEM)	M (SEM)	M (SEM)	M (SEM)		
Age	21.11 (0.33)	20.64 (0.29)	20.33 (0.37)	20.71 (0.3)	*F*(111,2) = 1.29	0.28
Sex	19 F/18 M	27 F/20 M	15 F/15 M	54 F/60 M	*χ* ^2^ = 0.76	0.69

Abbreviations: BSD, bipolar spectrum disorder; F/M, female/Male; *M*, mean; SEM, standard error of the mean.

### MRI acquisition and processing

All MRI data were collected on a 3T Verio MR scanner (Siemens, Erlangen, Germany) at Temple University. High resolution structural images were collected using a T1‐weighted image (sagittal plane; repetition time [TR] 1600 ms; echo time [TE] 2.46 ms; 176 interleaved slices; field of view [FOV] 250 mm; slice thickness = 1 mm, & flip angle 9°; See Supporting Information S1 for more information). FreeSurfer version 6.0 automatic segmentation software extracted surfaces (http://surfer.nmr.mgh.harvard.edu/; Fischl, 2012). MRI data were visually inspected for the quality of gray matter, consistent with Raamana et al. (2020). In particular, multiple individual raters examined each scan, and any errors were reviewed by KSFD and considered for manual edit, which was completed only if the error was verifiable in two planes of visualization (*n* = 23; 17.6%). There were no significant outliers in volumetric data for any of the frontostriatal or intracranial volumes. Individual surfaces were averaged using a non‐rigid, high‐dimensional spherical method that relies on the alignment of cortical folding patterns. mOFC and NAcc volumes were extracted for regions‐of‐interest analyses using the Desikan Atlas (Desikan et al., 2006).

### Analytic approach

Demographic features of the sample (including age, sex, and handedness) were compared across the BSD risk groupings, and reward sensitivity at the time of scan also was compared to confirm BSD risk groupings. In a single, repeated measures general linear model, mOFC and NAcc volumes (referred to collectively as frontostriatal volumes) were compared across BSD risk groups. This model examined sex and age at the time of the scan as a between‐subjects factor, given these factors may provide an important developmental context and may improve sensitivity to clinically relevant differences in gray matter volume (Nieuwenhuis et al., 2017). This approach allows us to examine a general, model‐corrected average of frontostriatal volumes (main effect of a variable on the multivariate outcome) and interactions by subregion for the mOFC and NAcc, correcting for the variance related to the other volume. Pairwise comparisons were corrected for multiple comparisons using Bonferroni correction.

Frontostriatal volumes related to the presence of a BSD were examined in follow‐up analyses that examined the relationship between volume scores and age at onset of first hypo/manic episode, age at onset of first depressive episode, and the number of lifetime BSD episodes. If a frontostriatal volume was related to a heightened risk for BSDs (i.e., high reward sensitivity but no BSD), then follow‐up analyses examined the relationship between volume scores and behavioral activation scores.

Intracranial volume (ICV) may distort analyses of sex differences (Voevodskaya et al., 2014) and reduce the accuracy of modeling sex by 60% (Sanchis‐Segura et al., 2020). Thus, it is optimal to present results without adjusting for ICV for analyses involving sex as a variable. However, we examined intracranial volume in two ways to ensure that it did not affect our analyses. First, in a separate model that parallels our primary analyses discussed above, we used the diagnostic group membership to predict ICV, accounting for variance related to sex and age. This analysis indicated that ICV did not vary by group (*t*(112) = 1.44, *b* = 0.12, *p* = 0.32). Second, ICV was included in follow‐up analyses; including ICV as a covariate did not change the magnitude or direction of the effect sizes. As a result, we present our results without intracranial volume in the models to maximize power and parsimony. Finally, to examine the specificity of our findings to the mOFC and NAcc, we examined a region‐of‐interest in the dorsal striatum (the putamen), for which we did not expect any group differences (Sherman et al., 2018).

## RESULTS

### Demographic characteristics and clinical symptoms

Analyses of variance (ANOVA) and chi‐square tests were used to examine group demographic differences across the BSD risk groups. There were no significant between‐group differences in age, *F*(2,111) = 1.29, *p* = 0.28, handedness, *F*(2,111) = 0.17, *p* = 0.68, or sex, *χ* ^2^(2) = 0.75, *p* = 0.69, Table 1 (see Table S4 for all clinical information). As expected from the study's recruitment strategy, groups significantly differed on the BAS‐T, *F*(2,110) = 29.41, *p* < .001, such that the low‐risk group scored significantly lower (*M* = 37.86, SEM = 0.96) than both the high‐risk (*M* = 43.72, SEM = 0.95), *p* < .001, and BSD groups (*M* = 43.94, SEM = 1.19), *p* < .001, Figure S1. Groups also were significantly different on SPSRQ‐SR scores, *F*(1,128) = 35.73, *p* < .001, such that the low‐risk group had significantly lower reward sensitivity (*M* = 10.80, SEM = 0.67) than the high‐risk (*M* = 16.85, SEM = 0.58), *p* < .001 and BSD groups (*M* = 17.33, SEM = 0.74), *p* < .001. There were no significant differences on BAS‐T, *p* = .10, and SPSRQ‐SR, *p* = 0.61 between the high‐risk and BSD groups.

### Frontostriatal gray matter volume analyses

In a general linear model, group (low‐risk, high‐risk, BSD), sex (male vs. female), and age were defined as between‐subjects factors to evaluate volumetric differences in frontostriatal volumes (mOFC, NAcc), which were nested within the subjects. There was a significant main effect of group on frontostriatal volumes (mOFC, NAcc), *F*(2,107) = 3.78, partial‐*ɳ* ^*2*^ = 0.066, *p* = 0.026, such that the BSD group displayed overall larger frontostriatal volumes (*M* = 6095.76, SEM = 116.67) compared to the low‐risk group (*M* = 5664.18, SEM = 104.92, Cohen's *d* (*d*) = 1.01, *p*_corrected_ = 0.02), but did not differ from the high‐risk group (*M* = 5828.61, SEM = 93.51, *d* = 0.42, *p*_corrected_ = 0.23). The low and high‐risk groups did not significantly differ on frontostriatal volume, *d* = 0.34, *p* = 0.74. There was a significant main effect of both age, *F*(1,107) = 6.63, *p* = .01, partial‐*ɳ* ^*2*^ = .058, and sex, *F*(1,107) = 21.61, *p* < 0.001, partial *ɳ* ^*2*^ = 0.168, such that older participants had a smaller frontostriatal volume (partial‐*r* = −0.23), and frontostriatal volume was larger in men than women (*d* = 1.83).

**FIGURE 1:**
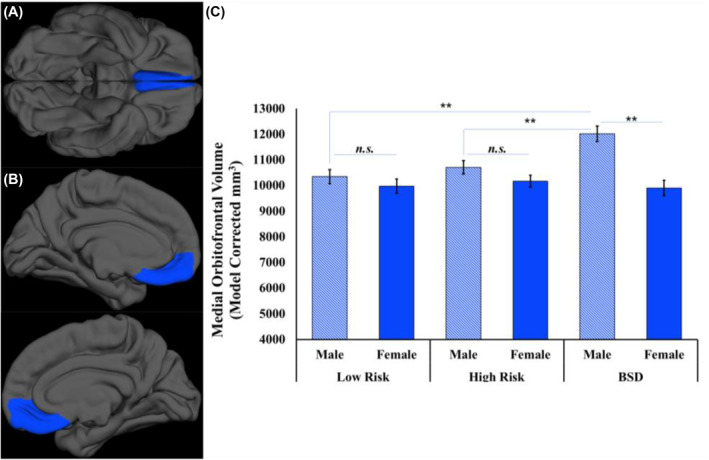
Medial orbitofrontal cortex gray matter volume by risk‐group status and sex: (A) displays the ventral view of the study sample average of the medial orbitofrontal cortex ROI on a study averaged surface front and, (B) displays a medial view of the study sample average of the medial orbitofrontal ROI on the average surface study sample (left on the top panel; right on the bottom panel), (C) a plot of the medial orbitofrontal cortical volume by group and sex with error bars displaying the standard error of the mean. BSD, Bipolar Spectrum Disorder; n.s., non‐significant. **p < 0.005

**FIGURE 2:**
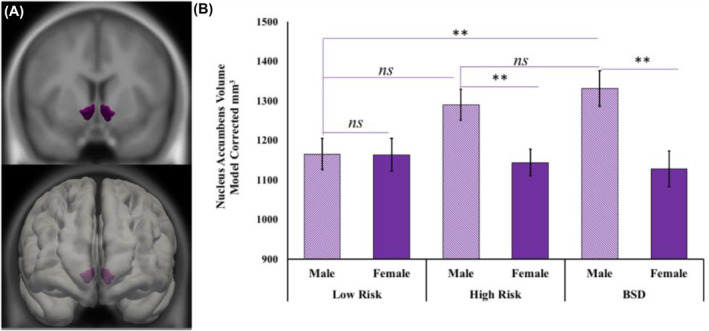
Nucleus accumbens volume by risk‐group status and sex: (A) Displays a group average of the nucleus accumbens (purple), which is shown in reference to a study average brain on the left and within the study averaged surface on the right, the top is the coronal view, and the bottom displays the left coronal view, (B) model corrected average of nucleus accumbens volume (mm^3^) is plotted by group and sex with error bars displaying the standard error of the mean. BSD, Bipolar Spectrum Disorder; n.s., non‐significant. **p < 0.005

These main effects were qualified by a region by group by sex interaction, *F*(2,107) = 5.18, *p* = .007, partial *ɳ* ^*2*^ = 0.088. Among males, those with a BSD had a significantly larger mOFC volume (*M* = 12,021.21, SEM = 305.58) than both the low‐risk (*M* = 10,347.68, SEM = 271.73, *d* = 1.01, *p*_corrected_ < .001) and high‐risk group participants (*M* = 10,710.62, SEM = 264.45, *d* = 0.74, *p*_corrected_ = 0.005, Figure 1). Males in the low and high‐risk groups did not differ on mOFC volume, *d* = 0.21, *p*_corrected_ = 0.99, Table 2. Also among males, those with a BSD had a significantly larger NAcc volume (*M* = 1331.83, SEM = 45.35) than the low‐risk group (*M* = 1164.94, SEM = 40.19, *d* = 0.68, *p*_corrected_ = 0.02), Figure 2. However, males in the high‐risk group (*M* = 1290.33, SEM = 39.11) did not differ from either the BSD (*d* = 0.16, *p*_corrected_ = 0.98) or low‐risk (*d* = 0.49, *p*_corrected_ = 0.086) groups on NAcc volume, Table 2. Simple main effects revealed that there were no group differences in either mOFC (*F*(2,107) = 0.287, *p* = 0.75) or NAcc (*F*(2,107) = 0.169, *p* = 0.85) volumes for females. All other interaction effects are available in Table 2.

**TABLE 2:** Bilateral medial orbitofrontal cortex and nucleus accumbens volume by risk‐group status and male and female participants

	Statistic	*p*‐value	Effect size		
Main effects					
Group	*F*(2,107) = 3.78	0.026	partial‐*ɳ* ^2^ = 0.066		
Low versus high‐risk		0.74	Cohen's *d* = 0.34		
Low‐risk versus BSD		0.02	Cohen's *d* = 1.01		
High‐risk versus BSD		0.23	Cohen's *d* = 0.42		
Age	*F*(1,107) = 6.63	0.011	partial‐*ɳ* ^2^ = 0.058		
Sex	*F*(1,107) = 21.61	<0.001	partial‐*ɳ* ^*2*^ = 0.168		
Male versus female			Cohen's *d* = 1.83		
Interaction effects					
Group by sex	(2,107) = 5.57	0.005	Partial‐*ɳ* ^2^ = 0.094		
Region by group	*F*(2,107) = 5.18	0.007	Partial‐*ɳ* ^2^ = 0.063		
Region by sex	*F*(2,107) = 17.45	<0.001	Partial‐*ɳ* ^2^ = 0.140		
Region by group by sex	*F*(2,107) = 5.18	0.007	Partial‐*ɳ* ^2^ = 0.088		

	Low‐risk	High‐risk	BSD	Low versus high‐risk	Low‐risk versus BSD	High‐risk versus BSD
	*n* = 37	*n* = 47	*n* = 30			
	*M* (SEM)	*M* (SEM)	*M* (SEM)	Cohen's *d*	Cohen's *d*	Cohen's *d*
Medial orbitofrontal cortex						
Males*	10,347.68 (271.73)	10,710.62 (264.45)	12,021.21 (305.58)	0.21	1.01*	0.74*
Females	9978.83 (280.15)	10,168.89 (33.65)	9901.115 (305.58)			
Nucleus accumbens						
Males*	1164.94 (40.19)	1290.33 (39.11)	1331.83 (45.35)	0.46	0.68*	0.16
Females	1164.27 (41.43)	1144.58 (33.65)	1128.90 (45.19)			

Abbreviations: *M*, mean; SEM, standard error of the mean; BSD, bipolar spectrum disorder.

*Bonferroni corrected *p*‐value < 0.05.

### Specificity analyses with putamen gray matter volume

In a general linear model, group (low‐risk, high‐risk, BSD), sex (male vs. female), and age were defined as between‐subjects factors to evaluate volumetric differences in the putamen. There were no significant differences by group (*F*(1,107) = 1.58, *p* = 0.40) or sex by group interaction (*F*(2,107) = 0.59, *p* = 0.15).

### Relationship to BSD clinical features

Follow‐up analyses were conducted within the BSD group to examine the relationship between mOFC and NAcc volumes and features of clinical course, including age at onset of BSD and number of BSD episodes, adjusting for sex. Later age at first hypo/manic episode was associated with larger NAcc, *r*(27) = 0.49, *p* = 0.01, and mOFC volumes, *r*(27) = 0.43, *p* = 0.03, but the total number of lifetime BSD episodes or age at first depression episode did not relate to frontostriatal volumes, *p'*s > 0.08.

## DISCUSSION

Enlarged frontostriatal (mOFC and NAcc) volumes were a corollary of BSD. An interaction revealed that this relationship varied by frontostriatal subregion (mOFC and NAcc). Individuals with a BSD displayed an enlarged mOFC compared to both individuals at high and low risk for BSD, suggesting an enlarged mOFC may reflect illness processes, progression, or onset, as opposed to a pre‐existent risk factor. This effect of the diagnostic group on the mOFC was driven by males with a BSD, providing preliminary evidence for sex‐specific mechanisms. We also observed sex‐specific effects for NAcc volume. Males with a BSD had an enlarged NAcc compared to males at low‐risk for BSD. Males in the high‐risk group, however, displayed an intermediate NAcc volume; they did not significantly differ from participants in either the low‐risk or BSD groups. Collectively, these findings are consistent with previous research on the volume of reward‐related brain regions in BSD but extend them to a behavioral risk design and consider sex‐specific effects in a developmental context.

Individuals with a BSD displayed an enlarged mOFC. The fact that only individuals with a BSD displayed an enlarged mOFC in this behavioral high‐risk design suggests that it reflects disease processes, progression, or onset, as opposed to a pre‐existent risk factor. These findings add to a growing body of evidence that abnormalities in the brain's reward circuitry contribute to the pathophysiology of bipolar disorder. The present study replicates research indicating that bipolar I and II disorder are characterized by an enlarged mOFC (Abe et al., 2016; Rimol et al., 2012). These findings also are consistent with the Reward Hypersensitivity Model, which argues that bipolar disorder, and in particular hypo/manic symptoms, are characterized by a hypersensitivity to rewarding stimuli in the environment (Alloy et al., 2016; Gruber & Johnson, 2009; Johnson, 2005; Johnson et al., 2012; Nusslock & Alloy, 2017; Nusslock et al., 2014). Further evidence that an enlarged mOFC may reflect BSD processes, progression, or onset is the fact that mOFC volume is related to age at first hypo/mania episode. These volumetric differences were only observed in male participants in the BSD group, highlighting the importance of considering sex‐specific development and presentation in clinical models of risk.

Individuals with a BSD also had a significantly enlarged NAcc volume compared to low‐risk individuals, consistent with previous research in BSD (Rimol et al., 2010). Extending prior work, our exploratory analyses suggest that an enlarged NAcc in BSDs may be specific to males. Further research is needed to examine whether these sex‐specific differences may reflect distinct neurodevelopmental processes (Urošević, Collins, Muetzel, Lim, et al., 2014) related to the impact of androgens on striatal neurodevelopment (Forbes et al., 2010) and may partially account for the sex‐specific presentation of increased mania in men (Goodwin & Jamison, 2007; Saunders et al., 2015; Sher et al., 2012). This specificity may reflect a unique risk trajectory for males with BSDs and highlights the importance of considering sex alongside clinical features of risk.

The males in the high‐risk group were intermediate on NAcc volume and did not significantly differ from males in either the low‐risk or BSD groups. This unexpected finding highlights several possibilities that should be considered in future research. First, individuals at high‐risk for BSD may truly display enlarged NAcc volume that is intermediate between those with a BSD and those at low risk for the disorder. Second, this finding may reflect the heterogeneity of the high‐risk group. The high‐risk group includes a mixture of individuals who will go on to develop a BSD and those who will not, which decreases sensitivity to relevant pathogenic processes. Finally, the volume of the NAcc may become more abnormal upon approaching BSD onset. The current study may be underpowered to detect such moderate effects. In fact, the difference in NAcc volume between the low and high‐risk groups was a medium effect size (*d* = 0.68). Given our small sample size, any interpretation must be made with caution. These possibilities emphasize the need for future longitudinal studies that assess NAcc volume across development and as a prospective predictor of BSD onset among high‐risk participants.

Although this study has several strengths, it's important for future research to address several limitations. The current study was designed to assess risk for BSD but was not explicitly designed to examine the interaction between sex and risk for BSD. As a result, our sex interaction findings should be interpreted cautiously. Future studies should recruit larger risk cohorts to examine sex differences within these cohorts. Such a study also would be better powered for exploratory whole‐brain analyses. Future studies also would benefit from a more comprehensive approach to sex and development by measuring features of puberty and hormones. Despite the utility of gray matter volume analyses, it is also possible that gray matter volume is less sensitive to gray matter abnormalities in females than males; future studies should also examine gray matter density (Gennatas et al., 2017). More research is needed on best practices in terms of examining sex‐specific biomarkers (Sanchis‐Segura et al., 2020; Voevodskaya et al., 2014). Future studies also should consider differences in symptomatology. In particular, men with a BSD tend to present with more mania, and women report high levels of depression (Goodwin & Jamison, 2007; Saunders et al., 2015), which may relate to sex specificity in gray matter volume. Relatedly, the present study included a range of BSD diagnoses, from cyclothymia to bipolar I disorder and a number of comorbid disorders. Although these milder forms of the illness and comorbidities may be a limitation, we hope that they improve the generalizability of findings to other BSD populations. Thus, collectively, more work is needed using diverse structural metrics, accounting for sex differences in symptom presentations, and among individuals with bipolar I disorder to better understand sex differences in frontostriatal brain structure in bipolar disorder.

## CONCLUSION

Individuals with a BSD displayed enlarged mOFC volume compared to both individuals at high and low risk for BSD, although this effect was observed in males only. This suggests that an enlarged mOFC reflects BSD processes, progression, or onset among males. By contrast, males with a BSD had a significantly enlarged NAcc volume compared to low‐risk individuals, whereas males in the high‐risk group were intermediate on NAcc volume and did not significantly differ from either the low‐risk or BSD groups. This pattern of findings suggests that NAcc volume may reflect an intermediary marker of risk for BSD for male individuals. However, future research needs to examine whether the volume of the NAcc enlarges as individuals approach BSD onset using large samples designed to assess sex differences and variation in symptom severity. Regardless, the present study highlights the importance of reward‐related brain structure in the pathophysiology of bipolar disorder but suggests that abnormalities in frontostriatal structure may apply more to men than women. Collectively, these findings highlight the importance of taking into consideration sex differences in future research on BSD and neurodevelopment.

## CONFLICT OF INTEREST

The authors have declared that they have no competing or potential conflicts of interest.

## ETHICAL CONSIDERATIONS

The treatment of subjects related to the study discussed in the attached manuscript was in accordance with the ethical guidelines of the APA and all relevant institutional review boards. None of the authors have any conflict of interest in the conduct and reporting of the enclosed research.

## AUTHOR CONTRIBUTIONS

**Katherine S. F. Damme:** Conceptualization (lead); formal analyses (lead); writing – original draft (lead); writing – review and editing (equal). **Lauren B. Alloy:** Funding acquisition (equal); project administration (lead); methodology (equal); supervision (equal); writing – review and editing (equal). **Nicholas J. Kelley:** Conceptualization (supportive); formal analyses (supportive); writing – review and editing (equal). **Ann Carroll:** Data Curation (equal); writing – review and editing (equal). **Christina B. Young:** Data Curation (equal); writing – review and editing (equal). **Jason Chein:** Supervision (supporting); project administration (supporting); writing – review and editing (equal). **Tommy H. Ng:** Investigation (equal); data Curation (equal); writing – review and editing (equal). **Madison K. Titone:** Investigation (equal); data Curation (equal); writing – review and editing (equal). **Corinne P. Bart:** Investigation (equal); data Curation (equal); writing – review and editing (equal). **Robin Nusslock:** Conceptualization (supporting); funding acquisition (equal); methodology (equal); supervision (equal); writing – review and editing (equal).

## PUBLIC HEALTH SIGNIFICANCE STATEMENT

Frontostriatal volumes were enlarged in bipolar spectrum disorder; this effect showed regional specificity and preliminary evidence of sex‐specificity to males. These findings emphasize the potential moderating role of sex in biomarkers of bipolar spectrum disorders.

## Supporting information

Supporting Information S1

## Data Availability

Data and statistical syntax are archived and will be made available upon request to the corresponding author and principal investigators (Robin Nusslock, Lauren Alloy).
