# Supplementary material for: Bipolar spectrum disorders are associated with increased gray matter volume in the medial orbitofrontal cortex and nucleus accumbens
Source: JCPP Adv. 2022 Mar 8;2(1):e12068. doi: 10.1002/jcv2.12068 (PMC9879263; doi:10.1002/jcv2.12068)
Supplement: Supplementary file 1 — Supporting Information S1 [file JCV2-2-e12068-s001.docx]

**Supporting Information**

**High-Risk Participant Recruitment.** Participants were recruited from a large, ongoing longitudinal project that prospectively identifies bipolar spectrum disorders. In the longitudinal study, participants were recruited via a multi-step selection procedure: two-stages of screening and a longitudinal phase (Alloy, Bender, et al., 2012). In the first step, 9,991 students (ages 14-19) from the Philadelphia area, completed two measures: the Behavioral Inhibition System/Behavioral Activation System scales (BIS/BAS; Carver and White, 1994) and the Sensitivity to Punishment/Sensitivity to Reward Questionnaire (SPSRQ; Torrubia, Ávila, Moltó, & Caseras, 2001). These measures were used to define a moderate reward sensitivity (Low-Risk) and high reward sensitivity (High-Risk) group. Participants who represent a statistical average (between the 40th to 60th percentile) on the Total BAS subscale of the BIS/BAS scales and the Reward subscale of the SPSRQ were classified as moderate reward sensitivity and considered low-risk for BSD (n = 750). Participants scoring in the 85th to 100th percentile on both measures were classified as high reward sensitivity, high-risk for BSD (n = 1,200). From this initial screening, 539 individuals (334 high reward and 205 moderate reward) returned for the second diagnostic screening. In the second stage of screening, participants completed a semi-structured diagnostic interview, including the expanded Schedule for Affective Disorders and Schizophrenia- Lifetime interview (exp-SADS-L; Endicott & Spitzer, 1978; Alloy, Bender, et al., 2012). Participants were excluded based on the presence of a primary psychotic disorder (n = 7) and poor fluency in English (n = 5). Finally, to begin the longitudinal phase, participants were invited to complete a baseline assessment, which included questionnaires, behavioral tasks, and interviews. At baseline, the study included 486 participants (300 high reward and 176 moderate reward). Participants enrolled in this study complete regular prospective assessments at approximately 6-month intervals over a period of up to five years.

**Participants.** A total of 130 young adults (52% female) completed the MRI portion of this study, who ranged in age from 18-27.

Table S1. Study Sample Race and Ethnicity

| Race | n | % |
| --- | --- | --- |
| African-American/Black | 30 | 23.1% |
| Asian-American/Asian | 12 | 9.2% |
| Biracial/Multiracial | 7 | 5.4% |
| Caucasian/White | 75 | 57.5% |
| Native American | 1 | 0.8% |
| Other | 4 | 3.1% |
| Not reported | 1 | 0.8% |
| Ethnicity | n | % |
| Hispanic/Latino | 7 | 5.4% |
| Not Hispanic/Latinx | 112 | 86.2% |
| Not reported | 11 | 8.5% |

This sample included 44 Low-Risk individuals, 53 High-Risk individuals, and 34 individuals with a bipolar spectrum diagnosis (BSD). In line with recruitment goals of the high-risk design, the Low-Risk group has significantly lower reward sensitivity than both the High-Risk group and the BSD group on both the Sensitivity to Reward Subscale and BAS Total.

*Figure S1.* *Confirmation of Reward Sensitivity Difference: Mean BAS Total Score and Sensitivity to Reward Score Difference by Group at Time of Scan*


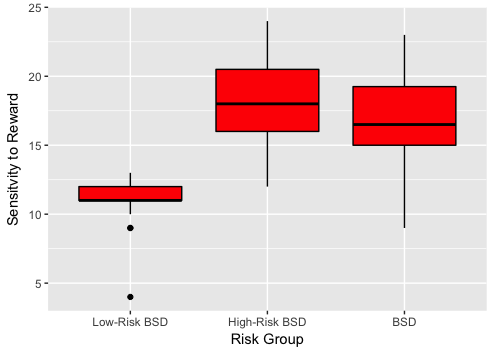

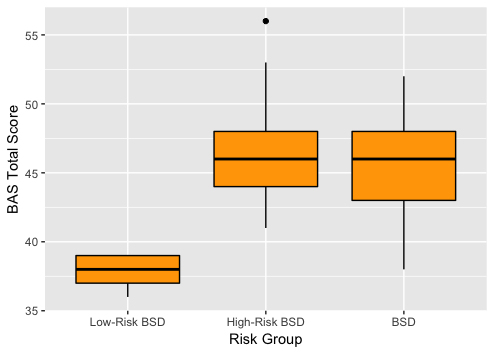


*Table S2.* *Risk Traits / Reward Sensitivity Across Groups*

| Reward Sensitivity | Low-Risk  *M(SEM)* | High-Risk  M(SEM) | BSD  M(SEM) | Group Comparison | *p-value* | |
| --- | --- | --- | --- | --- | --- | --- |
| BAS Total | 37.86 (1.09) | 43.72 (.95) | 46.03 (1.19) | *F*(110,2)= 15.3 | <.0001 | |
|  |  |  |  | Post-hoc | | |
|  |  |  |  | BSD vs High-Risk | | .94 |
|  |  |  |  | BSD vs Low-Risk* | | <.0001 |
|  |  |  |  | High-Risk vs Low-Risk* | | <.0001 |
|  |  |  |  | Group Comparison | *p-value* | |
| Sensitivity to  Reward Subscale | 10.81 (.67) | 16.85 (.59) | 17.33 (.73) | *F*(110,2)=29.41 | <.0001 | |
|  |  |  |  | Post-hoc | | |
|  |  |  |  | BSD vs High-Risk | | .61 |
|  |  |  |  | BSD vs Low-Risk* | | <.0001 |
|  |  |  |  | High-Risk vs Low-Risk* | | <.0001 |

Adjusting For Comorbidities

Although we excluded for any lifetime diagnoses of psychotic disorders, all groups may have lifetime diagnoses of unipolar depression, anxiety disorders, post-traumatic stress disorder, attention deficit hyperactivity disorder, substance use disorder, eating disorder, and/or obsessive compulsive disorder. In fact, there were non-BSD lifetime diagnoses present in every group: 26 Low-Risk individuals, 40 High-Risk individuals, and 20 BSD individuals. Across groups, there were no significant differences in the presence of non-BSD diagnoses, except unipolar depression and ADHD. There was a significantly higher occurrence of unipolar depression and ADHD in the High-Risk group than the Low-Risk group. Along with the presence of psychiatric diagnoses, there was medication use across all three groups as well. There was no significant difference across groups in the number of mood stabilizers (SSRIs, Tricyclics, Lithium, etc.) taken (Table 2. In terms of current diagnoses, there were current depression states across all three groups: 1 Low-Risk individual, 6 High-Risk individuals, and 4 BSD individuals. More details about the BSD group below (Table S3).

Table S3. Bipolar Spectrum Disorder Group Diagnoses and Course

| Diagnoses | | n |
| --- | --- | --- |
|  | Bipolar I | 4 |
|  | Bipolar II | 15 |
|  | Bipolar NOS | 3 |
|  | Cyclothymia | 8 |
|  | Current BSD | 5 |
|  | Lifetime BSD | 25 |
| Course and Disease Features | | *M* (*StD*) |
|  | Age at Hypo/Mania Onset | 18.04 (2.24) |
|  | Years since first Hypo/Mania Episode | 4.81 (2.97) |
|  | Number of Hypo/Mania Episodes | 1.44 (1.04) |

**MRI Data and Analysis.** All MRI data were collected on a 3T Verio MR scanner (Siemens, Erlangen, Germany) at Temple University. Structural images were collected in a T1-weighted anatomical image (sagittal plane; repetition time [TR] 1,600 ms; echo time [TE]2.46 ms; .5 mm3 isomorphic voxels, 176 interleaved slices; FOV 250mm; flip angle 9). FreeSurfer version 6.0 automatic segmentation software extracted surfaces (<http://surfer.nmr.mgh.harvard.edu/>; Fischl et al., 2012). Specifically, morphometric measurements were obtained by reconstructing representations of the gray/white matter boundaries. Individual surfaces were averaged using a non-rigid, high-dimensional spherical method that relies on the alignment of cortical folding patterns. Using cortical folding patterns generates a study specific space that maximizes the accuracy of the morphological alignment of homologous cortical locations based on individually defined anatomical landmarks, while minimizing metric distortion. Frontal lobe analyses compared gray and white matter volume extracted from Freesurfer v 6.0 defined frontal regions: lateral orbitofrontal, medial orbitofrontal, middle frontal (combining across rostral and dorsal regions), superior frontal, and frontal pole (Fischl, 2012). Initial analyses examined composite volume bilaterally as there was no strong hypothesis of laterality. Extracted brain volume, hemisphere laterality, lifetime diagnoses of MDD, and lifetime diagnoses of ADHD was included in follow up analyses and did not impact the magnitude or the direction of the findings and was therefore, removed from final analyses as the relationship between changing gray matter volume and extracted brain volume is unclear. Post-hoc analyses were conducted to examine the impact of a comorbid diagnosis, which did not significantly contribute to the model, *F*(1,106)=0.70, *p*=.40, but did not impact the significance of the other diagnoses.

*Table S4.* *Lifetime Diagnosis Group Comparison*

| Diagnosis | Low-Risk | High-Risk | BSD | Total | χ^2^ | p-value |
| --- | --- | --- | --- | --- | --- | --- |
| Any Diagnoses (non-BSD) | 26 | 40 | 20 | 86 | 3.81 | 0.14 |
| Subthreshold Depression | 7 | 4 | 1 | 12 | 4.61 | 0.10 |
| Unipolar Depression* | 10 | 23 | 0 | 33 | 21.42 | p<.0001* |
| Any Depression* | 17 | 27 | 1 | 45 | 29.02 | p<.0001* |
| Anxiety Disorder | 8 | 14 | 11 | 33 | 1.85 | 0.396 |
| PTSD | 2 | 1 | 0 | 3 | 1.97 | 0.37 |
| ADHD | 0 | 4 | 1 | 5 | 3.68 | 0.09 |
| Substance Use Disorder | 7 | 8 | 9 | 24 | 2.01 | 0.37 |
| Eating Disorder | 1 | 2 | 3 | 6 | 2.01 | 0.36 |
| OCD | 0 | 1 | 1 | 2 | 1.13 | 0.57 |

Included above are the counts for each group on the presence or absence of the diagnosis. PTSD – Post Traumatic Stress Disorder, ADHD - Attention Deficit Hyperactivity Disorder, BSD – Bipolar Spectrum Disorder, OCD = Obsessive Compulsive Disorder
